# Label Noise Learning Method for Metallographic Image Recognition of Heat-Resistant Steel for Use in Pressure Equipment

**DOI:** 10.3390/ma15197037

**Published:** 2022-10-10

**Authors:** Zhiyuan Shen, Haijun Hu, Ziyi Huang, Yu Zhang, Yafei Wang, Xiufeng Li

**Affiliations:** 1 School of Chemical Engineering, Xi’an Jiaotong University, Xi’an 710049, China; 2 SINOPEC Research Institute of Safety Engineering Co., Ltd., Qingdao 266000, China; 3 School of Computer Science, Shaanxi Normal University, Xi’an 710119, China; 4 China Special Equipment Inspection and Research Institute, Beijing 100029, China

**Keywords:** heat-resistant steel, metallographic image recognition, deep learning, label noise learning

## Abstract

In metallographic examination, spherular pearlite gradation, an important step in a metallographic examination, is the main indicator used to assess the reliability of heat-resistant steel. Recognition of pearlite spheroidization via the manual way mainly depends on the subjective perceptions and experience of each inspector. Deep learning-based methods can eliminate the effects of the subjective factors that affect manual recognition. However, images with incorrect labels, known as noisy images, challenge successful application of image recognition of deep learning models to spherular pearlite gradation. A deep-learning-based label noise method for metallographic image recognition is thus proposed to solve this problem. We use a filtering process to pretreat the raw datasets and append a retraining process for deep learning models. The presented method was applied to image recognition for spherular pearlite gradation on a metallographic image dataset which contains 422 images. Meanwhile, three classic deep learning models were also used for image recognition, individually and coupled with the proposed method. Results showed that accuracy of image recognition by a deep learning model solely is lower than the one coupled with our method. Particularly, accuracy of ResNet18 was improved from 72.27% to 77.01%.

## 1. Introduction

**Figure 1:**
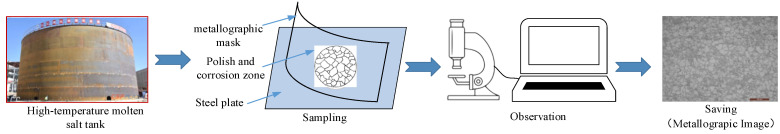
Diagram of metallographic examination procedure.

High-pressure equipment is widely used in the oil, chemical, and power industries. It has been reported that 4,396,300 pressure vessels and 1,012,600 km of high-pressure pipelines are currently in use in China [1]. Chromium-molybdenum steel is one of the main materials used to manufacture pressure vessels and high-pressure pipelines. The normal microstructures of this steel are ferrite and pearlite. However, pearlite spheroidization will occur in chrome-molybdenum steel after long-term exposure to a high-temperature environment, and this results in material degradation or even structural failure. Therefore, spherular pearlite gradation is an important evaluation indicator used to judge the performance of chrome-molybdenum steel [2,3]. In practice, the gradation of spherular pearlite is determined by metallographic examination, which is a microscopic examination method used to establish the microstructures and damage modes of metal materials, as illustrated in Figure 1.

The number of metallographic examination tasks that must be performed are increasing as the amount of high-temperature high-pressure equipment in use continues to grow. However, manual metallographic examination is a low-efficiency technique and is unable to meet the requirements of massive examination tasks. Computer vision methods have been applied successfully to facial image recognition [4] and object detection [5] and can also be extended to industrial testing applications. The CNPC Tubular Goods Research Institute [6] designed a metallographic structure analysis system based on digital image processing that involves image preprocessing, image segmentation, and pattern recognition. Deep learning-based methods are also receiving increasing attention from researchers [7]. Convolutional neural networks (CNNs; a type of deep learning model) have been applied to metallographic image recognition tasks, including microstructural segmentation [8], defect detection [9], and microstructure classification [10].

High-quality data and high-performance models are key factors in the successful application of deep learning methods. However, label noise in the datasets is a considerable problem for practical machine learning [11]. In practical tasks of industry defects classification, datasets always have incorrect labels. It is very hard or is not productive for data scientists to correct noisy labels manually. Damage assessment from metallographic examination of heat-resistant steel is mainly dependent on the subjective perceptions and experience of the individual inspector. In practice, therefore, some metallographic images will be wrongly labeled as damage images. For example, images of pearlite spheroidization are almost always incorrectly classified. Manual labeling of the data is essential for automatic recognition methods such as deep learning. Incorrectly labeled inputs, which are called noisy samples, thus represent obstructions to the construction of recognition models. Noisy samples in the training dataset will interfere with the real mapping between instances and their classes and will thus seriously degrade the performance of the deep learning model [12]. The influence of such noisy samples on deep learning models can be weakened by applying label noise learning methods, which can be divided into explicit methods and implicit methods [13]. The explicit methods focus on controlling the inputs to the model during the training process by removing or correcting the noisy samples in the datasets [14,15,16]. The implicit methods focus on construction of robust models for the noisy data [17,18,19,20]. Works of this type always build models based on noisy training sets and evaluate these models on clean test sets, thus ignoring the fact that such clean test sets do not exist in practical tasks. Noisy datasets are realistic and thus the accuracy of the models described above cannot be reliable.

When the accuracy of a model based on a noisy distribution approaches its maximum, the model will then approach the global optimum [21]. Therefore, evaluation of recognition models using noisy test datasets is a reliable approach. In this study, we propose a label noise learning method for metallographic image recognition of heat-resistant steel for use in high-pressure equipment. Incorrectly labeled metallographic images are filtered out gradually, and the number of images is controlled using a filter threshold. The influence of this threshold on the accuracy of CNN models is determined via a classification experiment performed on the cassava leaf disease dataset [22]. A case study of metallographic images of pearlite spheroidization is then performed.

## 2. Methods

### Negative Impact of Label Noise

Grades of material spheroidization are estimated by inspectors. However, these estimation processes are always affected by the subjective judgments of these inspectors. Therefore, automatic recognition techniques have become an important approach to elimination of the influence of these subjective factors during manual determination of the spheroidization grades [3]. Deep learning models require manually labeled data for training to allow them to obtain optimal parameters. Pearlite spheroidization grades can be divided into grades 1–5 based on metallographic images [2]. Sometimes, however, the classification is wrong or may be confused because these grades are assessed manually. Therefore, noisy labels may appear in the metallographic images dataset. This causes the problem of label noise, which can reduce the accuracy of a deep learning model greatly [23].

Automatic gradation of pearlite spheroidization is an N-class classification task. We use D to denote the unknown distribution and obtain an input dataset $S_{n}=\left\{\left(x_{1},y_{1}\right),\left(x_{2},y_{2}\right),\ldots ,\left(x_{N},y_{N}\right)\right\}\subset D$. The learning objective is to determine the best mapping function f:X→Y that can be obtained from the deep learning models. The parameters of the deep learning models are defined as *θ*. A loss function l is used to show the error of these models. The empirical risk over the distribution above is given as follows.

(1) $$R_{l,D}=\frac{1}{N}\overset{N}{\underset{i=1}{\sum }}l\left(f_{\theta }\left(x_{i}\right),y_{i}\right)$$

The optimal parameters *θ* are obtained when the empirical risk reaches a minimum.

(2) $$\theta =\arg minR_{l,D}$$

The label noise means that the correct input (image) corresponds to the wrong output (grade). In practice, we always obtain noisy data denoted by ${\tilde{S}}_{n}=\left\{\left(x_{1},{\tilde{y}}_{1}\right),\left(x_{2},{\tilde{y}}_{2}\right),\ldots ,\left(x_{N},{\tilde{y}}_{N}\right)\right\}\in \tilde{D}$. The optimal model parameters $\tilde{\theta }$ are then given by

(3) $$\tilde{\theta }=\arg min{\tilde{R}}_{l,D}$$

Obviously, $\theta \neq \tilde{\theta }$. Considerable effort must thus be made to determine θ from ${\tilde{S}}_{n}$.

**Figure 2:**
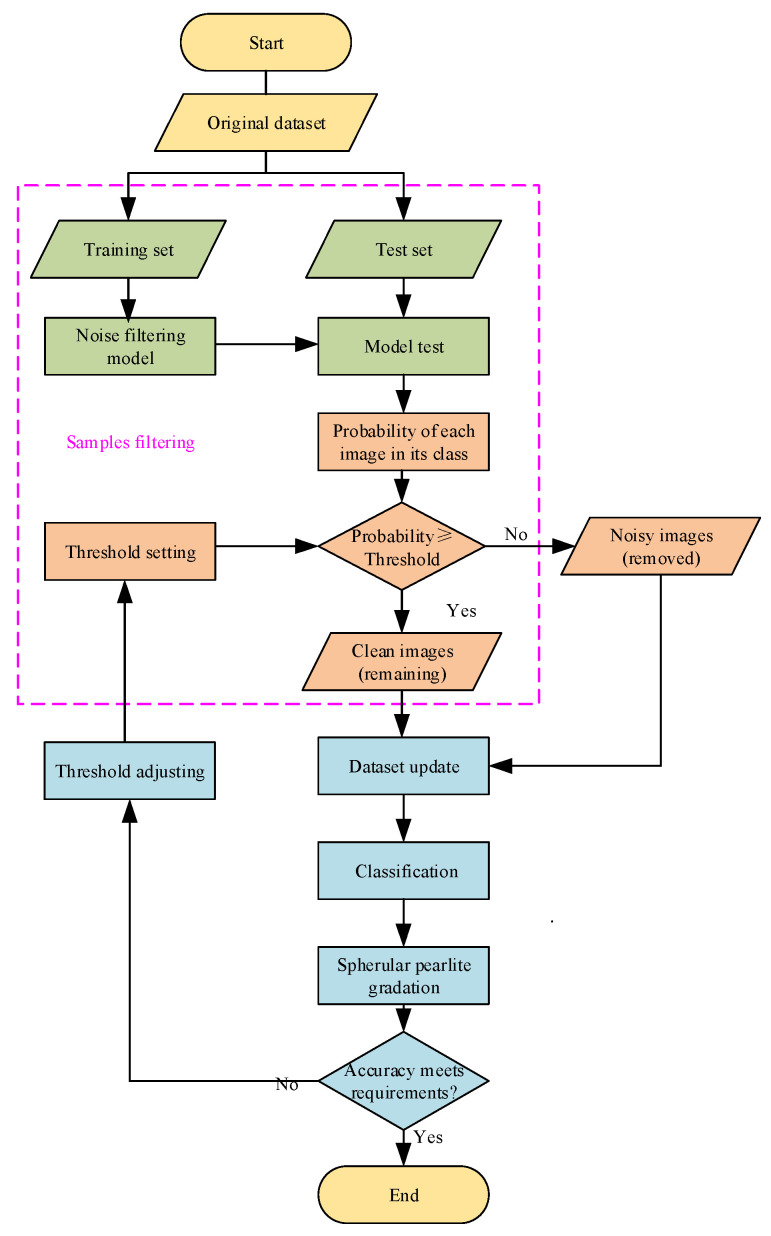
Process flowchart for the proposed method.

Reducing the influence of noisy samples on the model training process is essential for label noise learning. To delete the samples that are most likely to have been wrongly labeled, we added a filtering process for the datasets and a retraining process for the models. Models that were trained on raw datasets were used to filter out the noisy images, and these models were then retrained on the remaining datasets to obtain the classification models. A gradation of spherular pearlite by metallographic image recognition is an image classification task. CNNs are used as classification models in this paper. However, the datasets would normally be too small to train deep learning models if too many samples were deleted. A filter threshold was thus proposed to control the number of samples that were deleted. The optimum filter threshold was determined based on the prediction results of the classification models. The steps of the proposed method are illustrated in Figure 2.

The clean dataset $S_{n}$ was split into a clean training set $P_{k}$ and a test set $Q_{k}$ by K-fold cross-validation.

(4) $$S_{n}=\overset{K}{\underset{k=1}{\sum }}U_{n,k},\;P_{k}=S_{n}-U_{n,k},\;\;Q_{k}=U_{n,k}$$

**Figure 3:**
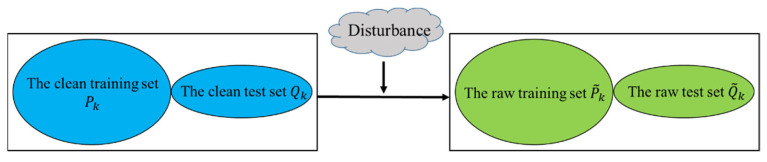
Relationship between the clean sets and the raw sets.

We split the raw noisy dataset ${\tilde{S}}_{n}$ via K-fold cross-validation to obtain the noisy training set ${\tilde{P}}_{k}$ and the noisy test set ${\tilde{Q}}_{k}$. The relationship between $S_{n}$ and ${\tilde{S}}_{n}$ is illustrated in Figure 3.

(5) $${\tilde{S}}_{n}=\overset{K}{\underset{k=1}{\sum }}{\tilde{U}}_{n,k},\;{\tilde{P}}_{k}={\tilde{S}}_{n.}-{\tilde{U}}_{n,k},\;{\tilde{Q}}_{k}={\tilde{U}}_{n,k}$$

The noise filtering model was obtained based on the noisy training set and test set.

(6) $${\tilde{M}}_{k}=f_{{\tilde{\theta }}_{k}}\left({\tilde{P}}_{k}\right)$$

The real class of the nth sample in the test set is *c*. The noise filtering model was used to output the probability that the sample is in class *c*.

(7) $$prob_{k,n,c}=softmax\left(f_{{\tilde{\theta }}_{k}}\left({\tilde{Q}}_{k}\right)\right)$$

A filter threshold v was set and was then compared with the probability.

(8) $$re_{n,th}=\left\{\begin{array}{c} 1,prob_{k,n,c}\geq v,v\in \left(0,1\right)\\ 0,prob_{k,n,c}<v,v\in \left(0,1\right) \end{array}\right.$$

For the nth sample, if $re_{n,th}=1$, then it would be retained; otherwise it would be removed. The remaining set $Q_{k,v}^{*}=U_{q,k,v}^{*}$, which contained q samples, was obtained after sample filtering.

**Figure 4:**
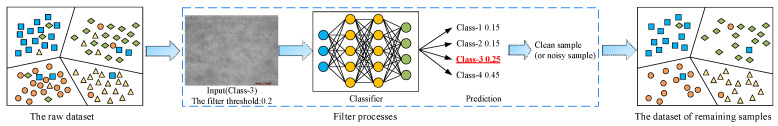
Process to filter out noisy samples.

As shown in Figure 4, when a sample is input, deep learning models will output its probability in each class. If the probability of the sample being in its real class is greater than or equal to the filtering threshold, the sample is considered to be a clean sample and retained. Otherwise, the sample is removed from the raw dataset since it is considered to be a noisy sample.

We obtain a remaining dataset $S_{q,v}^{*}$, a remaining training set $P_{k,v}^{*}$, and a remaining test set $Q_{k,v}^{*}$ using this approach. They are given as,

(9) $$S_{q,v}^{*}=\overset{K}{\underset{k=1}{\sum }}Q_{k,v}^{*}=\overset{K}{\underset{k=1}{\sum }}U_{q,k,v}^{*}$$

(10) $$P_{k,v}^{*}=S_{q,v}^{*}-U_{q,k,v}^{*},\;Q_{k,v}^{*}=U_{q,k,v}^{*}$$

**Figure 5:**
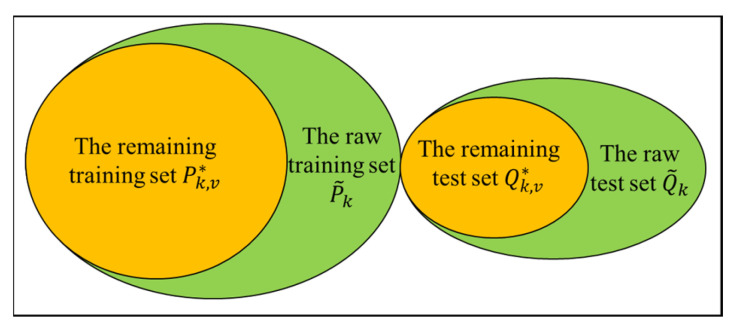
Relationship between the raw sets and the remaining sets. The remaining training set and the raw test set are used during retraining. The same test set is always used throughout the process.

The relationship between these datasets and the raw sets is shown in Figure 5.

The classification model $M_{k,v}^{*}$ can be obtained based on the remaining training set $P_{k,v}^{*}$ and the test set ${\tilde{Q}}_{k}$. During this process, only the training set was changed, while the test set remained the same.

(11) $$M_{k,v}^{*}=f_{\theta_{k}^{*}}\left(P_{k,v}^{*}\right)$$

We define Score as the accuracy that is obtained by ${\tilde{M}}_{k}\;$ and $M_{k,v}^{*}$ on the test set. It is given as,

(12) $$Score=\frac{1}{n_{samples}}\sum_{i=1}^{n_{samples}}1\left({\hat{y}}_{i}=y_{i}\right)$$

where ${\hat{y}}_{i}$ is the predicted value of the *i*-th sample, $y_{i}\;$ is the corresponding true value, and $1\left(x\right)$ is the indicator function [24].

The following relationships (Equation (13)) should be satisfied according to ref. [21], where ${\tilde{M}}_{k}$ is obtained based on the noisy dataset and $M_{k,v}^{*}$ is obtained based on the remaining dataset.

(13) $$Score\left(M_{k,v}^{*}\left({\tilde{Q}}_{k}\right)\right)>Score\left({\tilde{M}}_{k}\left({\tilde{Q}}_{k}\right)\right),Score\left(M_{k,v}^{*}\left(Q_{k}\right)\right)>Score\left({\tilde{M}}_{k}\left(Q_{k}\right)\right),\exists v\in \left(0,1\right)$$

A model that demonstrates a better performance than other models on a noisy distribution will also produce a better performance on a clean distribution. The optimal filter threshold can then be determined based on the accuracy of the model. It is given as,

(14) $$v=\arg max\left(score\left(M_{k,v}^{*}\left({\tilde{Q}}_{k}\right)\right)\right)$$

## 3. Results and Discussion

In the proposed method, the filter threshold is a decision variable and will affect the accuracy of the deep learning models. The threshold should thus be determined optimally before training commences.

Metallographic image recognition of spherular pearlite gradation is a fine-grained image classification task. A similarly fine-grained image classification task in the form of cassava leaf disease classification [22] was performed to determine how the filter threshold affects the CNN model accuracy. We selected 2000 images (two classes) from the total image dataset [22]. Noise labels were then created by exchanging some labels of their respective samples in two classes. Additionally, the label noise rate was set to vary from 10% to 40% in steps of 10%. The filter threshold was set to vary from 0.1 to 0.9 in steps of 0.1. ResNet18 was selected as the recognition model. Results of this analogue experiment are shown in Table 1. A model with higher classification accuracies on the noisy test set also raises higher classification accuracies on the clean test set. The filtering threshold can be determined to be the point at which the model accuracy on the noisy test set reaches a maximum.

### 3.1. Dataset of Pearlite Spheroidization Images

**Figure 6:**
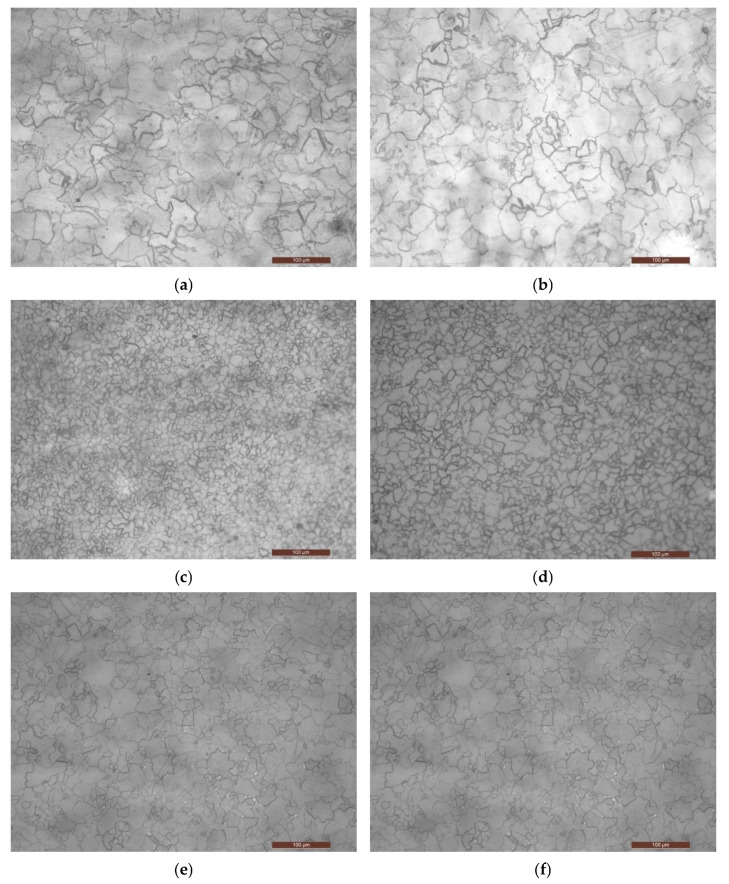
Problems with spherular pearlite gradation. Some images appear very similar but are labeled using different grades. (**a**) Normal; (**b**) Grade-5; (**c**) Normal; (**d**) Grade-5; (**e**) Grade-4 (elbow); (**f**) Grade-5 (body).

The degradation of pearlite spheroidization could be divided into 5 grades according to a related standard [2]. Pearlite spheroidization of grade-1 and grade-2 has little impact on the safety and reliability of pressure equipment [2]. A dataset of pearlite spheroidization was built which contained 422 metallographic images of heat-resistant steel. Thus, pearlite spheroidization of grade -1 and grade-2 is regarded as normal. In the dataset, 107 images are labeled as normal, 115 images are labeled as grade-3, 89 images are labeled as grade-4, and 111 images are labeled as grade-5 (see Table 2 and Figure 6). The filter threshold was set to vary from 0.1 to 0.5 in steps of 0.1.

### 3.2. Training Details

The parameters used for this experiment are given in Table 3. The learning rate would be reduced to one half of the previous learning rate if the model loss did not decrease for three consecutive epochs. Data augmentation [25] was performed during model training in forms including blurring, flipping, and cropping. Image normalization was performed during model testing. ResNet18 [26], EfficientNet-B0 [27], and RepVGG-A2 [28] were selected to verify the universality of the proposed method. In all experiments, only the training set was changed.

Take ResNet18 as an example. The implementation includes the following steps:

(1) The dataset of spherular pearlite gradation was divided into five subsets. One subset was taken as the raw test set and the others were treated as the raw training sets. ResNet18 was trained on the raw training sets. After being well trained, it is regarded as the baseline model.
(2) A filter process was carried out to remove noisy samples from the raw test set. Some remaining subsets were obtained by five-fold cross-validation. ResNet18 was retrained on the remaining subsets. If well retrained, it is used as the classification model.
(3) Comparisons between the baseline model and the classification model were carried out to check whether the filter-retraining process is valuable.

It should be mentioned that the test set was not changed during training and retraining steps. Thus, comparisons between trained-ResNet18 and retrained-ResNet18 are fair.

### 3.3. Results and Discussion

**Figure 7:**
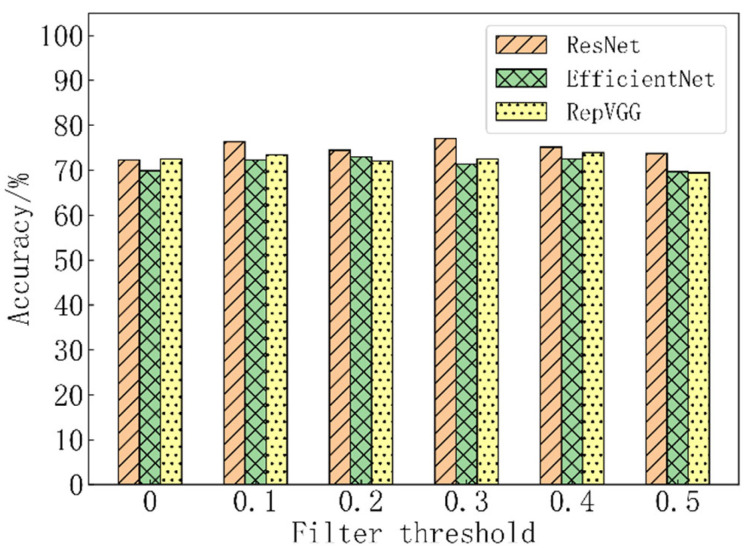
Variation of model accuracy with filter threshold.

The models that were obtained from regular training were regarded as the baseline models. The models that were obtained after image filtering and retraining were regarded as the classification models. The accuracy values for these models are given in Table 4 and Figure 7. For example, the initial accuracy of the ResNet18 (the baseline model) was 72.27%. When the filter threshold was set at 0.3, the accuracy of the ResNet18 (the classification model) improved from 72.27% to 77.01%, an increase of 4.74 percentage points. Similarly, when the filter threshold was set at 0.2, the accuracy of the EfficientNet-B0 improved from 69.91% to 72.99%. When the filter threshold was set at 0.4, the accuracy of the RepVGG-A2 network improved from 72.51% to 73.93%. The proposed method caused the deep learning models to learn and be optimized effectively on the noisy datasets.

Overall, the accuracy initially increased and subsequently decreased when the filter threshold increased. The results in Table 5 show that the image numbers decreased when the filter threshold increased. Therefore, the filter threshold is an upper-limited-type threshold. The value of the filter threshold should not be too large to ensure that the required recognition effect is realized. It is set to be less than 0.5 to ensure that more than 50% of the images are retained. The images in the training set can then represent the overall distribution of the dataset and reduce overfitting of the model.

The proposed method coupled with CNNs was compared with NTS [21] (NT: Noisy best teacher, NS: Noisy best student), which shows better performance than other label noise learning methods (GCE [18], Co-teaching [15], and DMI [29]) on noisy sets of CIFAR-10 [30] and CIFAR-100 [30]. Those CNNs that were obtained from regular training processes were regarded as baseline models. Experimental settings of NTS were the same as those in Section 3.2. The results are given in Table 6. The proposed method coupled with ResNet18 achieved an accuracy of 77.01% while NTS reached an accuracy of 73.70%. Predictions by baseline models were disturbed by noisy samples. NTS replaced the original labels of samples in datasets with labels predicted by baseline models. Thus, some labels incorrectly predicted are noisy labels and result in a limited model accuracy.

On the other hand, samples whose original labels are not as same as predicted labels are partially retained in the training set by our method and are helpful for our model to learn correct mapping relations between metallographic images and their grades.

## 4. Conclusions

In this study, a label noise learning method coupled with deep learning models for metallographic image recognition is proposed that benefits deep learning models by aiding in the learning of the correct mapping from incorrectly labeled samples of spherular pearlite gradation used in practical inspection tasks. We used a filtering process to pretreat the raw datasets and appended a retraining process for deep learning models. Additionally, a filter threshold was proposed to control the remaining image count. The proposed method effectively suppresses the negative influence of noise samples on model training.

First, an analogue experiment on cassava leaf disease classification was performed to disclose how the filter threshold affects the accuracy of deep learning models.

Next, the proposed method was applied to spherular pearlite gradation by image. We created a dataset containing 4-level gradation and 422 images of pearlite spheroidization. Three CNN models, ResNet18, EfficientNet-B0, and RepVGG-A2, were used to perform image classification, individually and coupled with the proposed method. The proposed method effectively improved the accuracy of deep learning models by using optimal filtering thresholds. The accuracy of ResNet18 was improved from 72.27% to 77.01% with a filter threshold of 0.4. In addition, the accuracy of EfficientNet-B0 was improved from 69.91% to 72.99%, and the accuracy of RepVGG-A2 was improved from 72.51% to 73.93%. Comparisons between our method and NTS were carried out. The proposed method coupled with ResNet18 reached an accuracy of 77.01% while NTS reached an accuracy of 73.70%.

Our work will be helpful in enabling deep learning models to learn real mappings from datasets with noisy labels in similar industry applications.

## Figures and Tables

**Table 1:** Accuracy of ResNet18 classification for cassava leaf disease with various noise rates.

Filter Threshold	Rates of Label Noise							
	10%		20%		30%		40%	
	Noisy Test Set	Clean Test Set	Noisy Test Set	Clean Test Set	Noisy Test Set	Clean Test Set	Noisy Test Set	Clean Test Set
0	0.8610	0.9494	0.7686	0.9382	0.6662	0.9014	0.5640	0.7732
0.1	0.8672	0.9576	0.7614	0.9310	0.6642	0.8922	0.5652	0.7684
0.2	0.8696	0.9580	0.7658	0.9414	0.6662	0.9082	0.5658	0.7582
0.3	0.8718	0.9610	0.7764	0.9528	0.6724	0.9148	0.5678	0.8038
0.4	0.8732	0.9632	0.7736	0.9532	0.6836	0.9340	0.5792	0.8104
0.5	**0.8752**	**0.9648**	0.7772	0.9564	**0.6848**	**0.9384**	**0.5892**	**0.8420**
0.6	0.8732	0.9620	**0.7796**	**0.9596**	0.6814	0.9286	0.5832	0.8376
0.7	0.8738	0.9630	0.7762	0.9502	0.6778	0.9214	0.5432	0.6840

**Table 2:** Image numbers for spherular pearlite gradation with each class.

Classes	Explanation	Numbers
Normal (Grade-1 and Grade-2)	Grade-1 and Grade-2 mean that pearlite spheroidization has not occurred.	107
Grade-3	Grade-3 means mild pearlite spheroidization.	115
Grade-4	Grade-4 means moderate pearlite spheroidization.	89
Grade-5	Grade-5 means serious pearlite spheroidization.	111

**Table 3:** Experimental settings for spherular pearlite gradation.

Parameters	Settings
Input image size	1000 × 750
Batches	30
Batch size	16
Initial learning rate	$3e^{-4}$
Optimizer	Adam
GPU	Nvidia GeForce RTX 3090

**Table 4:** Model accuracy values for spherular pearlite gradation with each filter threshold (%).

Filter Threshold	Accuracy		
	ResNet18	EfficientNet-B0	RepVGG-A2
0	72.27	69.91	72.51
0.1	76.30	72.27	73.46
0.2	74.41	**72.99**	72.04
0.3	**77.01**	71.33	72.51
0.4	75.12	72.51	**73.93**
0.5	73.70	69.67	69.43
0.6	72.27	71.80	71.56
0.7	69.91	67.77	65.88
0.8	70.14	67.06	63.51
0.9	64.69	65.88	62.80

**Table 5:** Image numbers for spherular pearlite gradation with each filter threshold.

Filter Threshold	Number		
	ResNet18	EfficientNet-B0	RepVGG-A2
0	422	422	422
0.1	377	366	382
0.2	352	342	360
0.3	327	318	328
0.4	307	301	295
0.5	293	284	257
0.6	263	272	223
0.7	228	257	196
0.8	200	239	171
0.9	156	211	128

**Table 6:** Comparison of the proposed method and NTS [21].

Methods	Accuracy		
	ResNet18	EfficientNet-B0	RepVGG-A2
Baseline	72.27	69.91	72.51
NTS	73.70	69.91	71.56
Ours	**77.01**	**72.99**	**73.93**

## Data Availability

The data presented in this study are available from the corresponding author upon reasonable request.
